# A systematic literature review of open source software quality assessment models

**DOI:** 10.1186/s40064-016-3612-4

**Published:** 2016-11-08

**Authors:** Adewole Adewumi, Sanjay Misra, Nicholas Omoregbe, Broderick Crawford, Ricardo Soto

**Affiliations:** 1 Covenant University, Ota, Nigeria; 2 Atilim University, Ankara, Turkey; 3 Pontificia Universidad Católica de Valparaíso, Valparaiso, Chile

**Keywords:** Analysis, Community, ISO 25010, Open source software, Quality assessment models

## Abstract

**Background:**

Many open source software (OSS) quality assessment models are proposed and available in the literature. However, there is little or no adoption of these models in practice. In order to guide the formulation of newer models so they can be acceptable by practitioners, there is need for clear discrimination of the existing models based on their specific properties. Based on this, the aim of this study is to perform a systematic literature review to investigate the properties of the existing OSS quality assessment models by classifying them with respect to their quality characteristics, the methodology they use for assessment, and their domain of application so as to guide the formulation and development of newer models. Searches in IEEE Xplore, ACM, Science Direct, Springer and Google Search is performed so as to retrieve all relevant primary studies in this regard. Journal and conference papers between the year 2003 and 2015 were considered since the first known OSS quality model emerged in 2003.

**Results:**

A total of 19 OSS quality assessment model papers were selected. To select these models we have developed assessment criteria to evaluate the quality of the existing studies. Quality assessment models are classified into five categories based on the quality characteristics they possess namely: single-attribute, rounded category, community-only attribute, non-community attribute as well as the non-quality in use models. Our study reflects that software selection based on hierarchical structures is found to be the most popular selection method in the existing OSS quality assessment models. Furthermore, we found that majority (47%) of the existing models do not specify any domain of application.

**Conclusions:**

In conclusion, our study will be a valuable contribution to the community and helps the quality assessment model developers in formulating newer models and also to the practitioners (software evaluators) in selecting suitable OSS in the midst of alternatives.

## Background

Prior to the emergence of open source software (OSS) quality models, the McCall, Dromey and ISO 9126 models were already in existence (Miguel et al. 2014). These models however did not consider some quality attributes unique to OSS such as community—a body of users and developers formed around OSS who contribute to the software and popularize it (Haaland et al. 2010). This gap is what led to the evolution of OSS quality models. Majority of the OSS quality models that exist today are derived from the ISO 9126 quality model (Miguel et al. 2014; Adewumi et al. 2013). It defines six internal and external quality characteristics, which are functionality, reliability, usability, efficiency, maintainability and portability. ISO 25010 replaced the ISO 9126 in 2010 (ISO/IEC 9126 2001), it has the following product quality attributes (ISO/IEC 25010 2001): functional suitability, reliability, performance efficiency, operability, security, compatibility, maintainability and transferability. The ISO 25010 quality in use attributes includes effectiveness, efficiency, satisfaction, safety and usability.

It is important to note that ISO 25010 can serve as standard for OSS only in terms of product quality and quality in use. It does not address unique characteristics of OSS such as the community. A key distinguishing feature of OSS is that it is built and maintained by a community (Haaland et al. 2010). The quality of this community also determines the quality of the OSS (Samoladas et al. 2008). From the literature, community related quality characteristics include (Soto and Ciolkowski 2009): maintenance capacity, sustainability, and process maturity. Maintenance capacity refers to the number of contributors to an OSS project and the amount of time they are willing and able to contribute to the development effort as observed from versioning logs, mailing lists, discussion forums and bug report systems. Furthermore, sustainability refers to the ability of the community to grow in terms of new contributors and to regenerate by attracting and engaging new members to take the place of those leaving the community. In addition, process maturity refers to the adoption and use of standard practices in the development process such as submission and review of changes, peer review of changes, provision of a test suite, and planned releases.

Since the advent of the first OSS quality model in 2003 (Adewumi et al. 2013), a number of other models have since been derived leading to an increasing collection of OSS quality models. Quality models in general can be classified into three broad categories namely: definition, assessment and prediction models (Ouhbi et al. 2014, 2015; Deissenboeck et al. 2009). Generally, OSS quality assessment models outline specific attributes that guide the selection of OSS. The assessment models are very significant because they can help software evaluators to select suitable OSS in the midst of alternatives (Kuwata et al. 2014). However, despite the numerous quality assessment models proposed, there is still little or no adoption of these models in practice (Hauge et al. 2009; Ali Babar 2010). In order to guide the formulation of newer models, there is need to understand the nature of the existing OSS quality assessment models. The aim of this study is to investigate the nature of the existing OSS quality assessment models by classifying them with respect to their quality characteristics, the methodology they use for assessment, and their domain of application so as to guide the formulation and development of newer models. Existing studies on OSS quality assessment models (Miguel et al. 2014; Adewumi et al. 2013) are largely descriptive reviews that did not seek to classify OSS quality assessment models along specific dimensions, or answer specific research questions. In contrast, this paper employs a methodical, structured, and rigorous analysis of existing literature in order to classify existing OSS quality assessment models and establish a template guide for model developers when they come up with new models. Thus, this study is a systematic literature review that investigates three research questions, namely: (1) what are the key quality characteristics possessed by the OSS assessment models? (2) What selection methods are employed for use in these assessment models? (3) What is the domain of application? In order to conduct this systematic review, the original guidelines proposed by Kitchenham (2004) have been followed.

The rest of this paper is structured as follows: “Methods” section describes the method of obtaining the existing OSS quality models. “Results” section presents the results obtained in the study, while “Summary and discussion” section discusses the findings of the study. “Conclusion and future work” section concludes the paper with a brief note.

## Methods

This section outlines the research questions posed in this study and also explains in detail the rationale behind each question. It goes on to discuss the search strategy for retrieving the relevant papers; criteria for including any given paper in the study; quality assessment of the retrieved papers as well as how relevant information was extracted from each selected paper.

### Research questions

This study aims at gaining insight into the existing OSS quality models and addresses three research questions. The three research questions alongside the rationale motivating each question is presented in Table 1. These form the basis for defining the search strategy.

**Table 1:** Research questions

Research question	Rationale
RQ1: What are the key quality characteristics possessed by the OSS quality assessment models?	To identify the most significant attributes possessed by the existing OSS quality assessment models in order to guide future proposals
RQ2: What are the methods used by these OSS quality assessment models for selection decisions	To identify the techniques used when applying the models to a selection scenario
RQ3: What is the domain of application of OSS quality assessment models?	To identify the software domains covered by the existing OSS quality assessment models, in addition to the targets of future studies

### Search strategy

A search string was defined based on the keywords derived from the research question as follows: “(Open Source Software OR libre OR OSS or FLOSS or FOSS) AND (model OR quality model OR measurement model OR evaluation model)”.

In order to retrieve the primary studies containing OSS quality models we made use of Scopus digital library. It indexes several renowned scientific journals, books and conference proceedings (e.g. IEEE, ACM, Science Direct and Springer). We considered only papers from (2003 to 2015) since the first OSS quality model emerged in 2003 (Haaland et al. 2010; Adewumi et al. 2013). We also focused on journal papers and conference proceedings in the subject area of Computer Science that were written in English. A total of 3198 primary studies were initially retrieved. After checking through their titles and abstracts, the number was reduced to 209. To be sure that no paper had been left out, we also performed a search in IEEE Explore, ACM and Springer using the same search string. No new papers were retrieved from this search that had not already been seen from the search in Scopus. Furthermore, a search was performed using Google Search and two relevant articles were retrieved (Duijnhouwer and Widdows 2003; Atos 2006) and added to make a total of 211 retrieved papers. These papers were read in detail to determine their suitability for inclusion.

### Inclusion criteria

Papers proposing cost models and conceptual models were removed. Also position papers and papers that did not present a model for assessing quality in OSS in order to guide selection in the midst of alternatives were also removed. A crosscheck was conducted through the reference list of candidate studies to ensure that no model had been left out. As a result, 19 primary studies were selected, which are further discussed in the next segment of this section.

### Quality assessment

Each primary study was evaluated by using the criteria defined in Adewumi et al. (2013). The criteria are based on four quality assessment (QA) questions:

QA1.: Are the model’s attributes derived from a known standard (this can be ISO 9126, ISO 25010 or CMMI)?
QA2.: Is the evaluation procedure of the model adequately described?
QA3.: Does a tool support the evaluation process?
QA4.: Is a demonstration of quality assessment using the model provided?

The questions were scored as follows:

QA1: Y (yes), the model’s attribute are mostly derived from a known standard, P (Partly), only a few of the model’s attributes are derived from a known standard; N (no), the model’s attributes are not all derived from a known standard.
QA2: Y, the evaluation procedure of the model are adequately described; P, the evaluation procedure was described inadequately; N, the evaluation procedure of the model was not described at all.
QA3: Y, the evaluation process is fully supported by a tool; P, the evaluation process is partially supported by a tool; N no tool support is provided for the evaluation process.
QA4: Y a complete demonstration of quality assessment using the model is provided; P only a partial demonstration of quality assessment using the model is provided; N there is no demonstration of quality assessment using the model provided.

The scoring procedure was Y = 1, P = 0.5, N = 0. The first author coordinated the quality evaluation extraction process. The first author assessed every paper, and assigned 5 papers each to the second, third and fourth authors and 4 papers to the fifth author so they could assess independently. When there was a disagreement, we discussed the issues until we reached agreement.

### Data extraction strategy

In this phase, the first author extracted the data while the other four authors checked the extraction. This approach though inconsistent with the medical standards summarized in Kitchenham’s guidelines (2004) has been found useful in practice (Brereton et al. 2007). The first author coordinated the data extraction and checking tasks, which involved all of the authors of this paper. Allocation was not randomized rather it was based on the time availability of the individual researchers. When there was a disagreement, we discussed the issues until we reached agreement.

The selected studies were gleaned to collect the data that would provide the set of possible answers to the research questions. Table 2 shows the data extraction form that was created as an Excel sheet and filled by the first author for each of the papers selected.

**Table 2:** Fields on the data extraction form

Study Refs.	
Title	
Classification	
Publication outlet	
Publication year	
(RQ1) Quality characteristics possessed	
(RQ2) Selection methods	
(RQ3) Application domain	

From Table 2 it can be observed that the information extracted includes: the Study Ref., title, and classification [publication outlet, publication year and research questions (RQ) 1, 2 and 3].

#### **RQ1.**

Quality characteristics that the models in the selected studies can possess include the product quality and the quality in use characteristics of the ISO 25010 namely: functional suitability, reliability, performance efficiency, operability, security, compatibility, maintainability, transferability, effectiveness, efficiency, satisfaction, safety and usability. We also include community related quality characteristics as described in the literature namely (Soto and Ciolkowski 2009): maintenance capacity, sustainability and process maturity.

#### **RQ2.**

The methods used by assessment models for selection can be classified as (Petersen et al. 2008; Wen et al. 2012):

- Data mining technique such as: Artificial Neural Network, Case-Based Reasoning, Data Envelope Analysis (DEA), Fuzzy Logic etc.
- Process: A series of actions, or functions leading to a selection result and performing operations on data
- Tool based technique: A technique that greatly employs software tools to accomplish selection task
- Model: A system representation that allows for selection based on investigation through a hierarchical structure
- Framework: A real or conceptual structure intended to serve as support or guide for selection process
- Other, e.g. guidelines

#### **RQ3.**

The domain of application can be classified as follows (Forward and Lethbridge 2008):

- Data dominant software—i.e. consumer-oriented software, business-oriented software, design and engineering software as well as information display and transaction entry
- Systems software—i.e. operating systems, networking/communications, device/peripheral drivers, support utilities, middleware and system components, software backplanes (e.g. Eclipse), servers and malware
- Control-dominant software—i.e. hardware control, embedded software, real time control software, process control software (e.g. air traffic control, industrial process, nuclear plants)
- Computation-dominant software—i.e. operations research, information management and manipulation, artistic creativity, scientific software and artificial intelligence
- No domain specified

### Synthesis method

The synthesis method was based on:

- Counting the number of papers per publication outlet and the number of papers found on a year-wise basis,
- Counting the primary studies that are classified in response to each research question,
- Presenting charts and frequency tables for the classification results which have been used in the analysis,
- Presenting in the discussion a narrative summary with which to recount the key findings of this study.

## Results

This section presents the results obtained in response to the research questions posed in this study. Table 3 is a summary of the OSS quality assessment models used in this study, their sources and year of publication. The first column of the table (Study Ref.) represents the reference number of each quality assessment model in ascending order. The table shows that 2009 has the most number of published papers—three publications in total. The year 2003, 2004, 2005 and 2012 have the lowest number of publications—one published paper each. All other years (2007, 2008, 2011, 2013, 2014, 2015) have two published papers.

**Table 3:** Summary of the OSS quality assessment models, their sources and year of publication

Study Refs.	Model name	Source	Year
Duijnhouwer and Widdows (2003)	OSMM	http://jose-manuel.me/thesis/references/GB_Expert_Letter_Open_Source_Maturity_Model_1.5.3.pdf	2003
Atos (2006)	QSOS	QSOS.org	2004
Wasserman et al. (2006)	Open BRR	Proceedings of the EFOSS Workshop	2005
Sung et al. (2007)	Sung et al.	Sixth International Conference on Advanced Language Processing and Web Information Technology	2007
Soto and Ciolkowski (2009)	QualOSS	Symposium on Empirical Software Engineering and Measurement	2009
Petrinja et al. (2009)	OMM	ICSE Workshop on Emerging Trends in Free/Libre/Open Source Software Research and Development	2009
Spinellis et al. (2009)	SQO-OSS	Electronic Notes in Theoretical Computer Science	2009
Aversano and Tortorella (2013)	EFFORT	Information and Software Technology	2013
Raffoul et al. (2008)	Raffoul et al.	International Conference on Software Engineering	2008
Alfonzo et al. (2008)	Alfonzo et al.	Australian Conference on Software Engineering	2008
Mathieu and Wray (2007)	Wray and Mathieu	AMCIS 2007 Proceedings	2007
Müller (2011)	Muller	International Digital Library Perspectives	2011
Chirila et al. (2011)	Chirila et al.	International Conference on Computational Intelligence and Informatics	2011
Raza et al. (2012)	OS-UMM	Computers in Human Behavior	2012
Adewumi et al. (2013)	Adewumi et al.	Interntional Confernce on Computational Science and Engineering	2013
Sudhaman and Thangavel (2015)	Sudhaman and Thangavel	International Jounal of Project Managemnt	2015
Sohn et al. (2015)	Sohn et al.	International Journal of Software Engineering	2015
Kuwata et al. (2014)	Kuwata et al.	Procedia Computer Science	2014
Sarrab and Rehman (2014)	Sarrab and Rehman	Advances in Engineering Software	2014

The studies were assessed for quality using the criteria described in the previous section (see “Quality assessment” section). The score for each study is shown in Table 4. The results of the quality analysis shows that all studies scored above 1 on the proposed quality assessment scale with only one study scoring less than 2. One study scored 4, five studies scored 3.5, five studies scored 3, five studies scored 2.5 and two studies scored 2.

**Table 4:** Quality evaluation of each article

Study Refs.	Model name	QA1	QA2	QA3	QA4	Total score
Duijnhouwer and Widdows (2003)	OSMM	Y	Y	N	Y	3
Atos (2006)	QSOS	P	Y	Y	N	2.5
Wasserman et al. (2006)	Open BRR	Y	Y	P	N	2.5
Sung et al. (2007)	Sung et al.	Y	Y	N	N	2
Soto and Ciolkowski (2009)	QualOSS	Y	P	N	N	1.5
Petrinja et al. (2009)	OMM	Y	P	P	N	2
Spinellis et al. (2009)	SQO-OSS	Y	Y	Y	P	3.5
Aversano and Tortorella (2013)	EFFORT	Y	Y	P	Y	3.5
Raffoul et al. (2008)	Raffoul et al.	Y	Y	P	Y	3.5
Alfonzo et al. (2008)	Alfonzo et al.	Y	Y	P	Y	3.5
Mathieu and Wray (2007)	Wray and Mathieu	Y	P	Y	Y	3.5
Müller (2011)	Muller	P	Y	N	Y	2.5
Chirila et al. (2011)	Chirila et al.	Y	Y	Y	N	3
Raza et al. (2012)	OS-UMM	Y	Y	N	Y	3
Adewumi et al. (2013)	Adewumi et al.	P	Y	N	Y	2.5
Sudhaman and Thangavel (2015)	Sudhaman and Thangavel	Y	Y	Y	Y	4
Sohn et al. (2015)	Sohn et al.	Y	P	N	Y	2.5
Kuwata et al. (2014)	Kuwata et al.	Y	Y	N	Y	3
Sarrab and Rehman (2014)	Sarrab and Rehman	Y	Y	N	Y	3

Table 5 shows the summary of the response to the research questions from each of the selected articles. From the table, it can be observed that an assessment model can belong to more than one category for RQ1 (an example is the assessment model in Study Ref. 8 which is single-attribute model, a non-community attribute model and a non-quality in use model).

**Table 5:** Summary of response to research questions from each article

Study Refs.	RQ1	RQ2	RQ3
Duijnhouwer and Widdows (2003)	Rounded category model	Process	Not specified
Atos (2006)	Single-attribute model, Non-community attribute model, Non-quality in use model	Process	Not specified
Wasserman et al. (2006)	Rounded category model	Process	Not specified
Sung et al. (2007)	Non-community attribute model	Model	Not specified
Soto and Ciolkowski (2009)	Non-quality in use model	Model	Not specified
Petrinja et al. (2009)	Non-quality in use model	Other	Not specified
Spinellis et al. (2009)	Rounded category model	Tool-based	Not specified
Aversano and Tortorella (2013)	Rounded category model	Framework	Data-dominant
Raffoul et al. (2008)	Non-community attribute model	Model	Data-dominant
Alfonzo et al. (2008)	Non-community attribute model	Model	Data-dominant
Mathieu and Wray (2007)	Single-attribute model Non-community attribute model	Data mining	Systems software
Müller (2011)	Rounded category model	Process	Computation-dominant software
Chirila et al. (2011)	Non-quality in use model Non-community attribute model	Tool-based	Not specified
Raza et al. (2012)	Single-attribute model Non-community attribute model	Framework	Not specified
Adewumi et al. (2013)	Non-quality in use model	Model	Computation-dominant software
Sudhaman and Thangavel (2015)	Single-attribute model Non-community attribute model	Data mining	Data-dominant
Sohn et al. (2015)	Rounded category model	Other	Data-dominant
Kuwata et al. (2014)	Community only attribute model	Other	Systems software
Sarrab and Rehman (2014)	Non-community attribute model	Model	Systems software

### RQ1. What are the key quality characteristics possessed by the models?

To address RQ1, we performed a comparative study of each identified model against ISO 25010 as well as community related quality characteristics described in “Background” section. Based on our comparative study, which is presented in Table 6, we classify the quality assessment models into five categories, which are discussed as follows:

**Table 6:** Comparative analysis

Quality characteristics	OSMM	QSOS	Open BRR	Sung et al.	QualOSS	OMM	SQO-OSS	EFFORT	Raffoul et al.	Alfonzo et al.	Wray and Mathieu	Muller	Chirila et al.	OS-UMM	Adewumi et al.	Sudhaman and Thangavel	Sohn et al.	Kuwata et al.	Sarrab and Rehman
*ISO 25010*																			
Product quality																			
Functional Suitability	x		x	x	x	x		x	x	x		x							x
Reliability	x				x	x	x	x	x				x		x				x
Performance efficiency	x		x		x	x		x									x		x
Operability	x		x	x	x				x	x									
Security	x		x	x	x	x	x		x				x						x
Compatibility	x			x	x	x			x	x									
Maintainability	x	x	x	x	x		x	x		x			x		x				x
Tranferability	x			x	x			x					x				x		
Quality in use																			
Effectiveness							x		x	x									
Efficiency											x	x				x			
Satisfaction																			
Safety																			
Usability	x		x	x				x	x	x		x		x			x		x
*Community related quality characteristics*																			
Maintenance Capacity	x		x		x	x	x	x							x		x	x	
Sustainability	x		x		x	x	x	x				x						x	
Process Maturity	x		x		x		x	x							x			x

1. Single-attribute models: This refers to models that only measure one quality characteristic. Qualification and Selection of Open Source software (QSOS) model (Atos 2006, Deprez and Alexandre 2008), Mathieu and Wray model (2007), Sudhaman and Thangavel model (2015) and Open Source Usability Maturity Model (OS-UMM) model (Raza et al. 2012) fall into this category. QSOS possesses maintainability as its quality characteristic. Mathieu and Wray as well as Sudhaman and Thangavel models both possess efficiency as their singular quality characteristic. In addition, OS-UMM possesses usability as its singular quality characteristic.
2. Rounded category models: This refers to models that possess at least one quality characteristic in each of the three categories used for comparison (i.e. product quality, quality in use and community related characteristics). Open Source Maturity Model (OSMM) (Duijnhouwer and Widdows 2003), Open Business Readiness Rating (Open BRR) model (Wasserman et al. 2006), Source Quality Observatory for Open Source Software (SQO-OSS) model (Samoladas et al. 2008; Spinellis et al. 2009), Evaluation Framework for Free/Open souRce projecTs (EFFORT) model (Aversano and Tortorella 2013), Muller (2011) and Sohn et al. model (2015) fall into this category of models. OSMM possesses all the quality characteristics in the product quality category as well as in the community-related quality characteristics but only possesses usability in the quality in use category. Open BRR and EFFORT models both possess all the community-related quality characteristics, some of the product quality characteristics and usability from the quality in use category. SQO-OSS possesses all the community-related quality characteristics, three of the product quality characteristics and effectiveness from the quality in use category. Muller model possesses one characteristic each from the product quality and community-related categories. It also possesses efficiency and usability from the quality in use category. As for Sohn et al. model, it possesses two quality characteristics from the product quality category and one quality characteristic each from the quality in use and community-related quality categories.
3. Community-only attribute model: This refers to a model that only measures community-related quality characteristics. The only model that fits this description is the Kuwata et al. model (2014) as seen in Table 6. The model does not possess any quality characteristic from the product quality or quality in use categories.
4. Non-community attribute model: This refers to models that do not measure any community-related quality characteristics. QSOS (Atos 2006), Sung et al. (2007), Raffoul et al. (2008), Alfonzo et al. (2008), Mathieu and Wray, Chirila et al. (Del Bianco et al. 2010a), OS-UMM (Raza et al. 2012), Sudhaman and Thangavel, and Sarrab and Rehman (Sarrab and Rehman 2014) models fall into this category.
5. Non-quality in use models: This refers to models that do not include any quality in use characteristics in their structure. QSOS (Atos 2006, Deprez and Alexandre 2008), QualOSS (Soto and Ciolkowski 2009), OMM (Petrinja et al. 2009, Del Bianco et al. 2010b, Del Bianco et al. 2011, Chirila et al. (2011), Adewumi et al. (2013), and Kuwata et al. models are the models in this category.

From our classification, it is possible for a particular model to belong to more than one category. QSOS for instance belongs to three of the categories (i.e. it is a single-attribute model, non-community attribute model and non-quality in use model). Mathieu and Wray model (2007), Chirila et al. model (2011), OS-UMM (Raza et al. 2012), Sudhaman and Thangavel model (2015), as well as Kuwata et al. model (2014) all belong to two categories respectively. Precisely, Mathieu and Wray model is a single-attribute model and non-community attribute model. Chirila et al. model is a non-community attribute model as well as a non-quality in use model. OS-UMM is a single attribute model and a non-community attribute model. Sudhaman and Thangavel model is both a single-attribute model and non-community attribute model. Kuwata et al. model is both a community-only attribute model and a non-quality in use model. All the other models belong to a single category and they include: OSMM (Duijnhouwer and Widdows 2003), Open BRR (Wasserman et al. 2006), Sung et al. (2007), QualOSS (Soto and Ciolkowski 2009), OMM (Petrinja et al. 2009), SQO-OSS (Samoladas et al. 2008), EFFORT (Aversano and Tortorella 2013), Raffoul et al. (2008), Alfonzo et al. (2008), Muller (2011), Adewumi et al. (2013), Sohn et al. as well as Sarrab and Rehman models (2014).

Table 6 is a comparative analysis between the OSS quality models presented in Table 3 and the ISO 25010 model. It also features community related characteristics and how they compare with the OSS quality models. Cells marked with ‘x’ indicate that the OSS quality model possesses such characteristic similar to ISO 25010. An empty cell simply means that the OSS quality model does not possess such characteristic as found in ISO 25010.

**Fig. 1:**
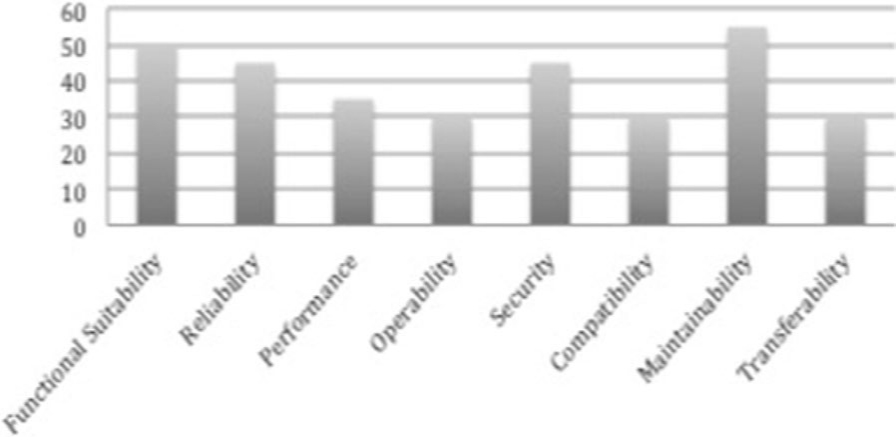
Frequency distribution of ISO 25010 product quality characteristics in OSS quality models

 Figure 1 shows the frequency distribution of the ISO 25010 Product quality characteristics in the OSS quality models we considered. It shows that maintainability is measured by 55% of the existing OSS quality models making it the most common product quality characteristic measured by existing OSS quality models. This is followed by functional suitability, which is measured in 50% of the existing quality models. The least measured are operability, compatibility and transferability that are each measured by 30% of the existing quality models. From Fig. 1, it can be inferred that the maintainability of a given OSS is of more importance than the functionality it possesses. This is because being an OSS; the code is accessible making it possible to incorporate missing features. However, such missing features can be difficult to implement if the code is not well documented, readable and understandable which are all attributes of maintainable code. Similar inferences can be made as regard the other quality characteristics. For instance, the reliability and security of an OSS can be improved upon if the code is maintainable. In addition, the performance efficiency, operability, compatibility and transferability can all be improved upon with maintainable code.

**Fig. 2:**
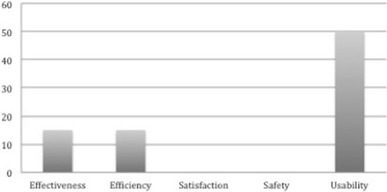
Frequency distribution of ISO 25010 quality in use characteristics in OSS quality models

Figure 2 shows the frequency distribution of the ISO 25010 Quality in Use characteristics in the OSS quality models we considered. It shows that usability is measured by 50% of the existing OSS quality models making it the most commonly measured characteristic in this category. It is followed by effectiveness and efficiency, which are both considered by 15% of the existing OSS quality models. Satisfaction and safety on the other hand are not considered in any of the existing OSS quality models. From Fig. 2, it can be easily inferred that usability is the most significant attribute under the quality in use category and hence all other attributes in this category add up to define it. In other words, usable OSS is one that is effective in accomplishing specific tasks, efficient in managing system resources, safe for the environment and provides satisfaction to an end-user.

**Fig. 3:**
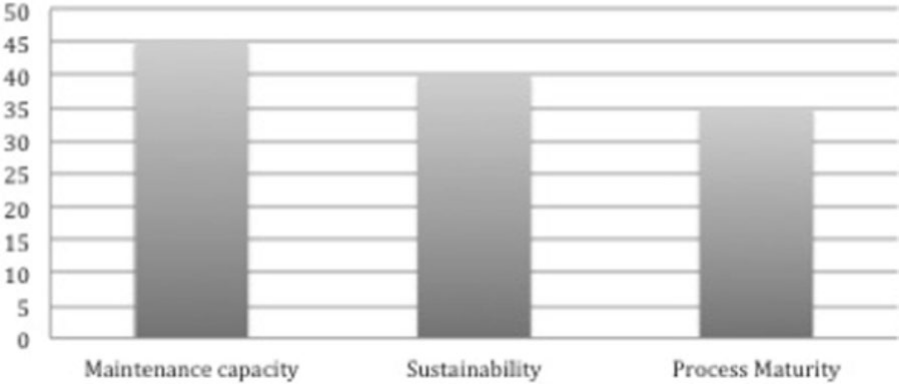
Frequency distribution of community related quality characteristics in OSS quality models

Figure 3 shows the frequency distribution of community related quality characteristics in the OSS quality models we considered. It shows that maintenance capacity is measured in 45% of the existing OSS quality models making it the most commonly measured attribute in this category. It is closely followed by sustainability that is measured by 40% of the existing OSS quality models. Process maturity is the least measured attribute in this category and is considered in 35% of the existing OSS quality models. It can be inferred from Fig. 3 that evaluators of an OSS quality via its community are mostly interested in the maintenance capacity of such a community in comparison to the sustainability of the community. Also, they are more concerned about the sustainability of the community than the maturity of the community’s processes.

### RQ2. What are the methods applied for reaching selection decisions?

**Fig. 4:**
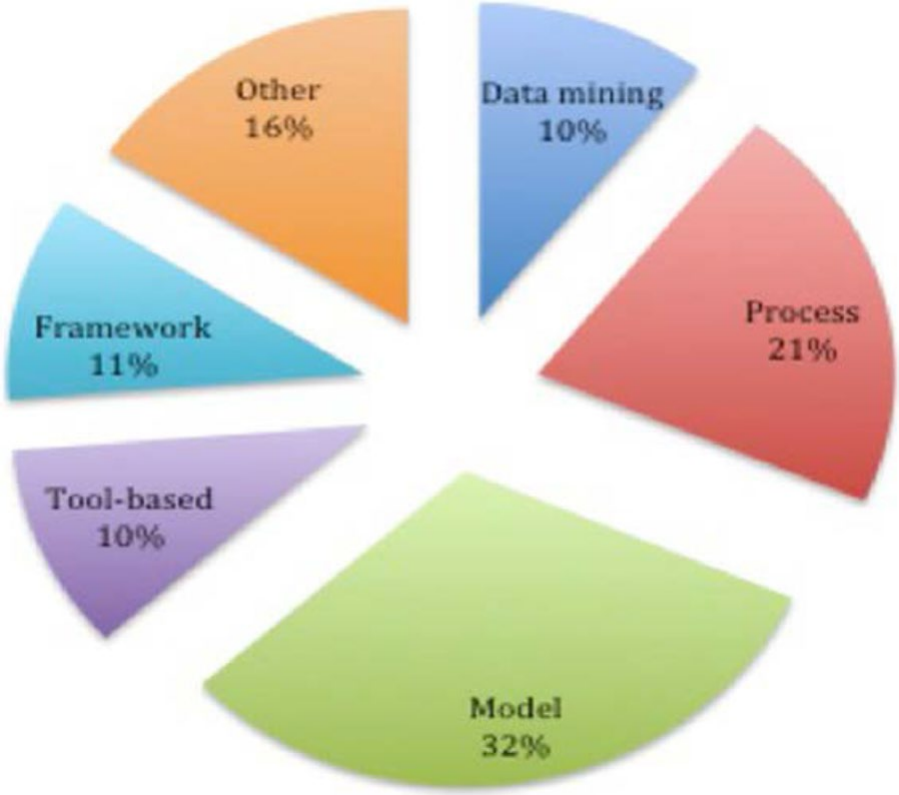
Selection methods used in OSS quality models

- Figure 4 depicts the various selection methods adopted in the existing OSS quality models for reaching a decision in the midst of alternatives. The model approach, which entails making system representation that allows for selection based on investigation through a hierarchical structure is the most common selection method used in the existing literature and is used by six (32%) of the existing models. This is followed by the process approach that accounts for use in 21% (four) of the existing models. For the “other” category, three (16%) of the models use a form of guideline in the selection process. Framework approach accounts for 11% while the data mining approach, as well as the tool-based approach both account for 10% each of the existing OSS quality models. In general, it can be observed that more emphasis is placed on non-automated approaches in the existing quality models and so applying these models in real life selection scenarios is usually time-consuming and requires expertise to conduct (Hauge et al. 2009; Ali Babar 2010).

### RQ3. What is the domain of application?

**Fig. 5:**
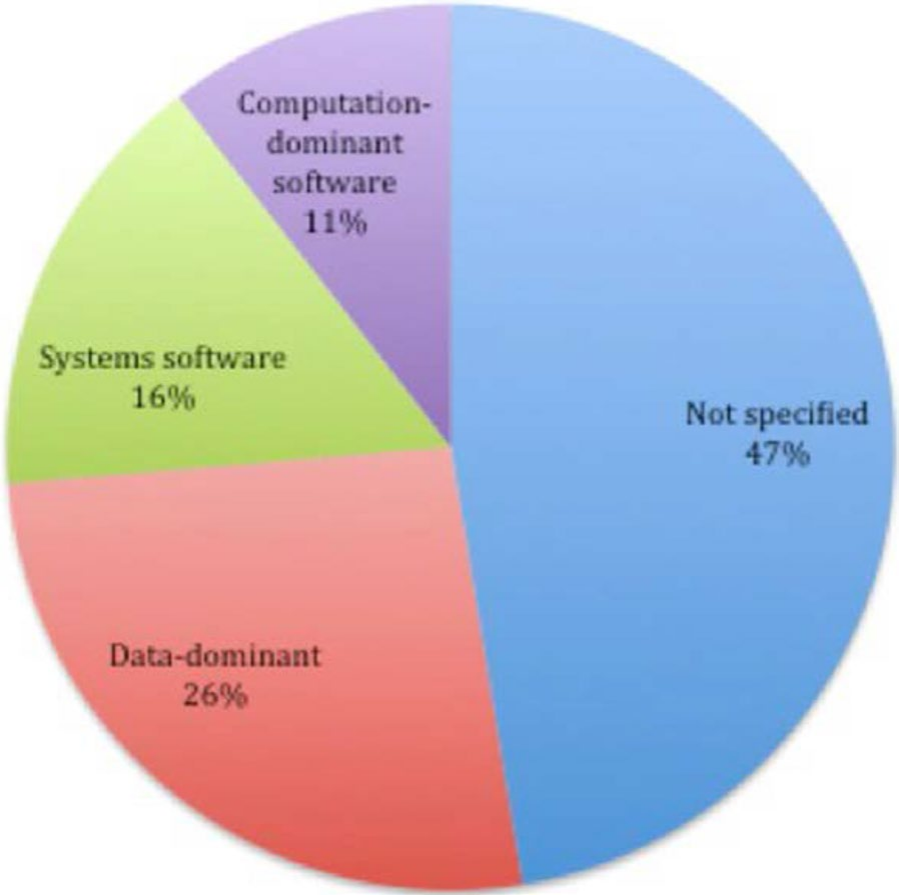
Domains in which OSS quality models have been applied

Figure 5 depicts the domain of application of the existing OSS quality assessment models. In general, majority of the models do not specify the domain of application. However, for those with specific domain of application, we observed that majority focus on measuring quality in data-dominant software that includes: business-oriented software such as Enterprise Resource Planning and Customer Relationship Management solutions; design and engineering software as well as information display and transaction systems such as issue tracking systems. System software evaluation accounts for 16% while computation-dominant software accounts for 11%.

## Summary and discussion

### Principal findings

- From the existing OSS quality models considered in this study, 20% of the models only measure a single quality attribute. Models in this category include: QSOS (which measures maintainability) (Atos 2006), Wray and Mathieu (Mathieu and Wray 2007) (which measures efficiency), OS-UMM (which measures usability) (Raza et al. 2012) and Sudhaman and Thangavel model (which measures efficiency) (Sudhaman and Thangavel 2015). Furthermore, 50% of the existing models do not measure community related quality characteristics even though community is what distinguishes OSS from their proprietary counterpart. Models in this category include: QSOS (Atos 2006), Sung et al. model (2007), Raffoul et al. model (2008), Alfonzo et al. model (2008), Wray and Mathieu model (Mathieu and Wray 2007), Chirila et al. model (2011), OS-UMM (Raza et al. 2012), Sudhaman and Thangavel model (2015) and Sarrab and Rehman model (2014). In addition, 35% of the models touch on all categories. They include: OSMM (Duijnhouwer and Widdows 2003), Open BRR (Wasserman et al. 2006), SQO-OSS (Spinellis et al. 2009), EFFORT (Aversano and Tortorella 2013), Müller model (2011) and Sohn et al. model (2015). Among these models a number of them have been applied to selection scenarios and reported in the literature. A notable example is the EFFORT model, which has been applied to evaluate OSS in the customer relationship management (CRM) domain (Aversano and Tortorella 2011) as well as in the enterprise resource-planning (ERP) domain (Aversano and Tortorella 2013).
- From the existing OSS quality models, it is observed that in the aspect of product quality as defined by ISO 25010, maintainability is the most significant quality characteristic; Usability is the most significant quality in use characteristic in the existing OSS quality models while Maintenance capacity is the most significant community related characteristic in the OSS quality assessment models. Also worthy of note is that satisfaction and safety attributes of quality in use are never considered in the OSS quality models.
- The model approach is the most adopted selection method in the existing OSS quality models. The least considered are the tool-based and data mining selection approaches. However, as newer publications emerge we expect to see other approaches and data mining gaining more ground.
- Majority (47%) of the existing models do not specify any domain of application. As for those with specific domain of application, a greater percentage focus of data-dominant software especially enterprise resource planning software. Computation-dominant software is the least considered in this regard. Software in this category includes: operations research, information management and manipulation, artistic creativity, scientific software and artificial intelligence software.
- From the this study, we also observed that none of the existing models evaluate all the criteria that we laid out, in terms of every quality characteristic under product quality, quality in use, and community related quality characteristics.

### Implications of the results

Based on the comparison of the existing quality assessment models, there is clearly no suitable model—each model has its own limitations. As a result, the findings of this analysis have implications especially for practitioners who work towards coming up with new assessment models. They should note the following points in line with the research questions posed in this study:

- Emphasis should shift from trying to build comprehensive models (containing all the possible software characteristics) to building models that include only essential quality characteristics. This study has shown that these essential quality characteristics include: maintainability, usability and maintenance capacity of software community. By narrowing down to these three essential quality characteristics, model developers would help to reduce the burden of OSS evaluation via existing quality assessment models, which has been referred to largely as being laborious and time consuming to conduct (Hauge et al. 2009; Ali Babar 2010).
- Newer models should incorporate selection methods that are amenable to automation as this is not the case in most of the existing OSS quality assessment models reviewed in this study. The selection methods mostly adopted are the model (32%), process (21%) and other (16%) such as guidelines, which are not easily amenable to automation (Fahmy et al. 2012). Model developers should thus turn their focus to data mining techniques (Leopairote et al. 2013), framework or tool-based selection methods, which are currently among the least considered options. The advantage this offers is that it will help quicken the evaluation process resulting in faster decision-making. Following this advice could also bring about increased adoption of the models in practice (Wang et al. 2013). In addition, model developers can also consider modeling quality assessment as a multi-criteria decision-making (MCDM) problem so as to facilitate automation as seen in recent studies (Fakir and Canbolat 2008; Cavus 2010, 2011). A MCDM problem in this context can be regarded as a process of choosing among available alternatives (i.e. different OSS alternatives) based on a number of attributes (quality criteria). Considering this option opens the model developer to several well-known MCDM methods that amenable to automation such as: DEA, Analytic Hierarchy Process (AHP), and Technique for Order of Preference by Similarity to Ideal Solution (TOPSIS) to mention a few (Zavadskas et al. 2014).
- From Fig. 5, it can be observed that 47% of the quality assessment models considered do not mention the domain of application. This implies that most of the models were designed to be domain-independent. As such, domain-independence should be the focus of model developers (Wagner et al. 2015). A domain independent model is one that is able to assess quality in various category of OSS including those that are data-dominant, system software, control-dominant and computation-dominant. It should also be able to this with little or no customization. By following this particular consideration, the model proposed can tend to be widely adopted and possibly standardized.

### Threats to validity

Construct threats to validity in this type of study is related to the identification of primary studies. In order to ensure that, as many relevant primary studies as possible were included, different synonyms for ‘open source software’ and ‘quality model’ were included in the search string. The first and second author conducted the automatic search for relevant literature independently and the results obtained were harmonized using a spreadsheet application and duplicates were removed. The reference sections of the selected papers were also scanned to ensure that all relevant references had been included. The final decision to include a study for further consideration depended on the agreement of all the authors. If a disagreement arose, then a discussion took place until consensus was reached.

Internal validity has to do with the data extraction and analysis. As previously mentioned, the first author carried out the data extraction of the primary studies and assigned them to the other authors to assess. The first author also participated in assessing all the primary studies and compared his results with those of the other authors and discrepancies in results were discussed until an agreement was reached. The assignment process of the primary studies to the other authors was not randomized because the sample size (number of primary studies) was relatively small and the time availability of each researcher needed to be considered. In order to properly classify the primary studies based on the quality characteristics they possessed, the authors adopted the ISO 25010 model (2001) as benchmark. All the authors were fully involved in the process of classifying the primary studies and all disagreements where discussed until a consensus was reached.

To mitigate the effects of incorrect data extraction, which can affect conclusion validity, the steps in the selection and data, extraction activity was clearly described as discussed in the previous paragraphs. The traceability between the data extracted and the conclusions was strengthened through the direct generation of charts and frequency tables from the data by using a statistical package. In our opinion, slight differences based on publication selection bias and misclassification would not alter the main conclusions drawn from the papers identified in this study.

As regards the external validity of this study, the results obtained apply specifically to quality assessment models within the OSS domain. Quality assessment models that evaluate quality in proprietary software are not covered. In addition, the validity of the inferences in this paper only concern OSS quality assessment models. This threat is therefore not present in this context. The results of this study may serve as starting point for OSS quality researchers to further identify and classify newer models in this domain.

## Conclusion and future work

The overall goal of this study is to analyze and classify the existing knowledge as regards OSS quality assessment models. Papers dealing with these models were identified between 2003 and 2015. 19 papers were selected. The main publication outlets of the papers identified were journals and conference proceedings. The result of this study shows that maintainability is the most significant and ubiquitous product quality characteristic considered in the literature while usability is the most significant attribute in the quality in use category. Maintenance capacity of an OSS community is also a crucial quality characteristic under community related quality characteristics. The most commonly used selection method is the model approach and the least considered are the tool-based and data mining approaches. Another interesting result is that nearly half (47%) of the selected papers do not mention an application domain for the models in their research. More attention should be paid to building models that incorporate only essential quality characteristics. Also, framework, tool-based and data mining selection methods should be given more attention in future model proposals.

This study could help researchers to identify essential quality attributes with which to develop more robust quality models that are applicable in the various software domains. Also, researchers can compare the existing selection methods in order to determine the most effective. As future work, we intend to model OSS quality assessment as a MCDM problem. This will afford us the opportunity to choose from a range of MCDM methods one (or more) that can be used to evaluate quality in OSS across multiple domains.

## Abbreviations

CRM: customer relationship management
DEA: Data Envelope Analysis
EFFORT: Evaluation Framework for Free/Open souRce projecTs
ERP: enterprise resource-planning
MCDM: multi-criteria decision making
Open BRR: Open Business Readiness Rating
OSMM: Open Source Maturity Model
OS-UMM: Open Source Usability Maturity Model
OSS: open source software
QA: quality assessment
QSOS: Qualification and Selection of Open Source software
RQ: research question
SQO-OSS: Source Quality Observatory for Open Source Software
TOPSIS: Technique for Order of Preference by Similarity to Ideal Solution
